# Evidence for a Xer/*dif* System for Chromosome Resolution in Archaea

**DOI:** 10.1371/journal.pgen.1001166

**Published:** 2010-10-21

**Authors:** Diego Cortez, Sophie Quevillon-Cheruel, Simonetta Gribaldo, Nicole Desnoues, Guennadi Sezonov, Patrick Forterre, Marie-Claude Serre

**Affiliations:** 1 Institut Pasteur, Unité Biologie Moléculaire du Gène chez les Extrêmophiles, Paris, France; 2 Institut de Biochimie et de Biophysique Moléculaire et Cellulaire, UMR8619-CNRS, Université Paris-Sud 11, IFR115, Orsay, France; 3 Université Pierre et Marie Curie, Paris, France; 4 Institut de Génétique et Microbiologie, Université Paris-Sud 11, UMR8621-CNRS, IFR115, Orsay, France; Agency for Science, Technology and Research, Singapore

## Abstract

Homologous recombination events between circular chromosomes, occurring during or after replication, can generate dimers that need to be converted to monomers prior to their segregation at cell division. In *Escherichia coli*, chromosome dimers are converted to monomers by two paralogous site-specific tyrosine recombinases of the Xer family (XerC/D). The Xer recombinases act at a specific *dif* site located in the replication termination region, assisted by the cell division protein FtsK. This chromosome resolution system has been predicted in most Bacteria and further characterized for some species. Archaea have circular chromosomes and an active homologous recombination system and should therefore resolve chromosome dimers. Most archaea harbour a single homologue of bacterial XerC/D proteins (XerA), but not of FtsK. Therefore, the role of XerA in chromosome resolution was unclear. Here, we have identified *dif*-like sites in archaeal genomes by using a combination of modeling and comparative genomics approaches. These sites are systematically located in replication termination regions. We validated our *in silico* prediction by showing that the XerA protein of *Pyrococcus abyssi* specifically recombines plasmids containing the predicted *dif* site *in vitro*. In contrast to the bacterial system, XerA can recombine *dif* sites in the absence of protein partners. Whereas Archaea and Bacteria use a completely different set of proteins for chromosome replication, our data strongly suggest that XerA is most likely used for chromosome resolution in Archaea.

## Author Summary

Bacteria with circular chromosome and active homologous recombination systems have to resolve chromosomal dimers before segregation at cell division. In *Escherichia coli*, the Xer site-specific recombination system, composed of two recombinases and a specific chromosomal site (*dif*), is involved in the correct inheritance of the chromosome. The recombination event is tightly regulated by the chromosome translocase FtsK. This chromosome resolution system has been predicted in most bacteria and further characterized for some species. Intriguingly, most archaea possess a gene coding for a recombinase homologous to bacterial Xers, but none have homologues of the bacterial FtsK. We identified the specific target sites for archaeal Xer. This site, present in one copy per chromosome, is located in the replication termination region and shows sequence similarities with bacterial *dif* sites. *In vitro*, the archaeal Xer recombines this site in the absence of protein partner. It has been shown that DNA–related proteins from Archaea and Eukarya share a common origin, whereas their analogues in Bacteria have evolved independently. In this context, Eukarya and Archaea would represent sister groups. Therefore, the presence of a shared Xer-*dif* system between Bacteria and Archaea illustrates the complex origin of modern DNA genomes.

## Introduction

In Bacteria, homologous recombination is essential during DNA replication to resume stalled replication forks and to repair DNA double and single strand breaks. Odd numbers of homologous recombination events between circular chromosomes generate dimers, which need to be resolved to ensure proper segregation in daughter cells. In *Escherichia coli* two paralogous site-specific tyrosine recombinases XerC and XerD were shown to convert chromosome dimers to monomers [1] by acting at a specific DNA recombination site, *dif*, located close to the replication termination region [2]–[4]. Homologues of XerCD are widespread in the bacterial domain, and *dif* sites have been characterized in several Proteobacteria and Firmicutes [5]–[10]. *dif* sites are semi-conservative inverted repeats formed by two arms (Xer protein binding sites) of 11 base pairs [11], separated by a spacer of 6 bp and are fairly conserved among Bacteria [5]. In Proteobacteria the XerCD activity is tightly regulated by the cell division protein FtsK, a DNA translocase anchored at the division septum [12]–[15]. In *E. coli*, 8 bp G-rich polar sequence elements (KOPS) direct FtsK translocation on DNA [16]–[19]. KOPS are oriented from the origin of replication towards *dif* where their polarity is precisely inverted. FtsK DNA translocation is therefore always oriented towards the *dif* site and *dif* sites carried on a chromosome dimer are brought together at midcell. FtsK further controls chromosome dimer resolution by activating XerD activity through protein-protein interactions [20], [21]. In several *Lactococcus* and *Streptococcus* strains, the canonical bacterial XerCD-*dif* system has been replaced by a single tyrosine recombinase, XerS (distantly related to XerCD) whose gene is located next to its specific *dif*-like site and localized at the terminus of replication [22]. Strikingly, this XerS-*dif*-like system still depends on the KOPS-oriented FtsK activity to form the synaptic complex for recombination [22].

Archaea harbour circular chromosomes and have an active homologous recombination system [23]. Therefore, they are expected to resolve chromosomal dimers to ensure proper chromosome segregation. It was previously reported that most archaeal genomes encode a single protein homologous to bacterial XerCD [24]; however, none encode a FtsK homologue. It is thus unclear whether archaeal Xer-like proteins (hereafter called XerA) are involved in chromosome resolution in Archaea, as in Bacteria.

In order to determine whether XerA is involved in chromosome resolution, we performed an *in silico* search for XerA specific recombination *dif*-like sites in four closely related archaeal genomes from *Thermococcales*. We identified a highly conserved 28 bp sequence that shares 14 out of 28 bases with characterized bacterial *dif* sites. The predicted *dif* sites are systematically located in the replication termination regions of the four genomes. The same analysis performed on three *Sulfolobales* genomes revealed that a similar site is also present in this crenarchaeotal species. We further identified short polarized sequences that point towards the predicted *dif* sites in *Thermococcales* genomes. We validated the *in silico* predictions by showing that a purified recombinant XerA protein from *Pyrococcus abyssi* specifically recombines plasmids carrying the predicted *dif* site of this archaeon. The recombination activity did not require the presence of any protein partner, in contrast to bacterial Xer-mediated recombination. Our data strongly suggest that XerA is most likely used for chromosome resolution in Archaea.

## Results

The majority (88%) of archaeal genomes sequenced so far (KEGG database [25]) harbour single orthologues of the bacterial XerCD recombinases. Alignments of several bacterial XerCD proteins with XerA proteins from different Archaea revealed that they share a conserved C-terminal domain where the catalytic site (Figure S1) is located. The six catalytic residues (R-K-H-R-[H/W]-Y) characteristic of tyrosine recombinases [26] are perfectly conserved in archaeal XerA proteins [24].

**Figure 1:**
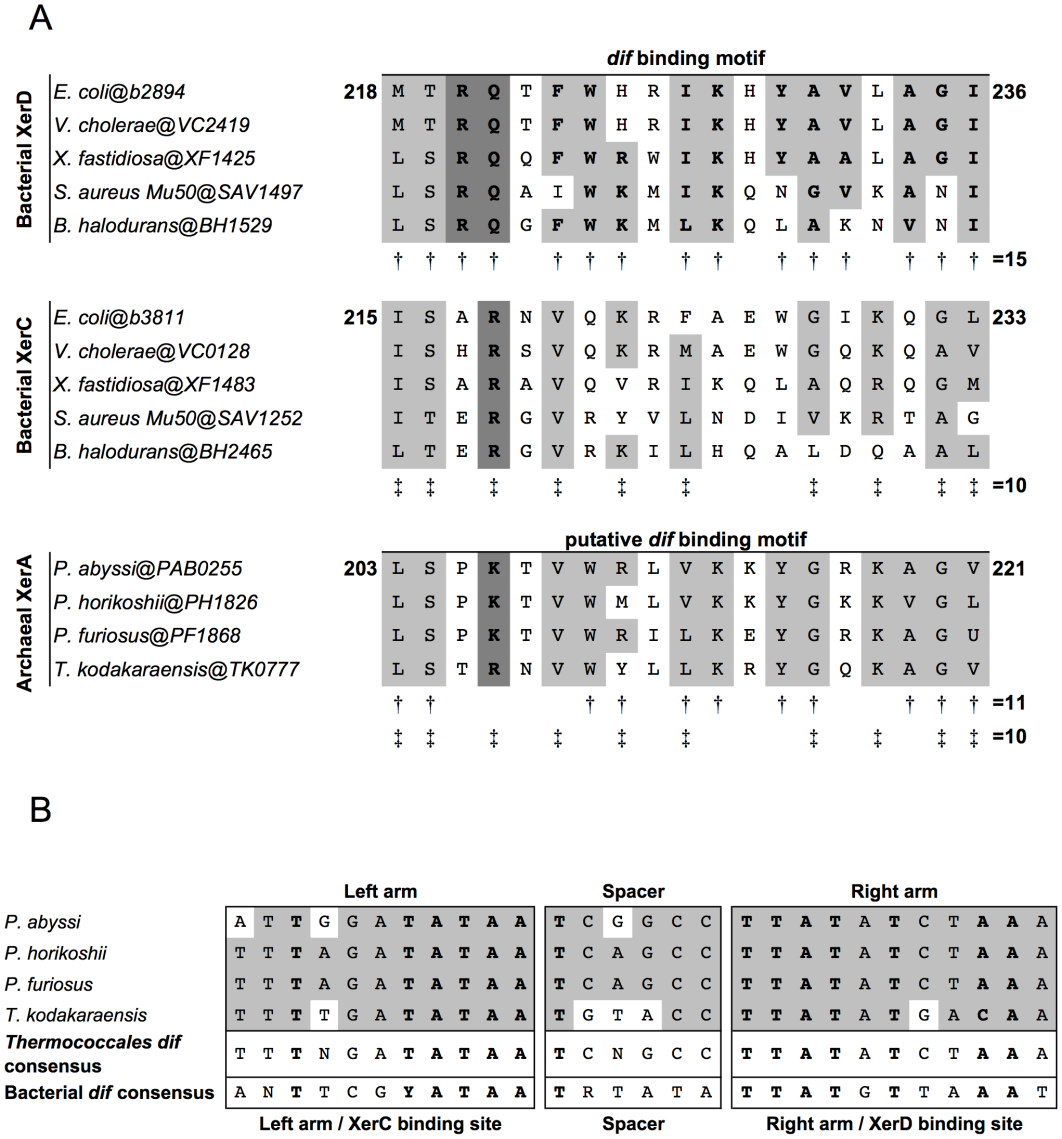
Alignments of Xer *dif*-binding motifs and *dif* sites from Archaea and Bacteria. A. Alignments of the *dif* binding motifs from bacterial XerC and XerD and archaeal XerA proteins. Amino acids shared between XerD *dif* binding motifs, XerC *dif* binding motifs and putative *dif* binding motifs of archaeal XerA proteins from *Thermococcales* are boxed. Conserved positions in XerD *dif* binding motifs are indicated by ‘†’ and conserved positions in XerC *dif* binding motifs are indicated by ‘‡’. The putative *dif* binding motifs of XerA proteins share several positions with XerD and/or XerC proteins. The key motif for each family (XRX for XerC, XKX for XerA and and RQX for XerD) are shown in bold and shaded in dark gray. B. Predicted *Thermococcales dif* sites are highly conserved, show very few or no mismatches between the two arms of the inverted repeat and share several positions with the bacterial *dif* consensus (bold letters). Consensus sequences are indicated according to the IUPAC code.

The more variable outer sequences of the bacterial *dif* sites are the place of specific amino-acids/bases contacts that drive protein-DNA interaction specificity. Several amino acids residues involved in these contacts were identified, which led to the definition of a *dif*-binding region within the C-terminal domain of the Xer recombinases [27]–[29]. The *dif* binding motif is also conserved in XerA proteins. Notably, the key residues that define binding specificity for the XerC or XerD binding sites are distinct from XerC or XerD in XerA (Figure 1A and Figure S1).

### *In silico* identification of *dif*-like sites in archaeal genomes

To search for conserved putative archaeal *dif* sites, we selected as first candidates four closely related genomes of *Thermococcales* since their XerA proteins [*Pyrococcus abyssi* (Pab0255), *Pyrococcus horikoshii* (PH1826), *Pyrococcus furiosus* (PF1868) and *Thermococcus kodakaraensis* (TK0777)] are the most similar to bacterial XerCD (35%–39% identity; Figure S2) among archaeal XerA. The putative *dif*-binding motif of these XerA proteins shares numerous conserved positions with both XerC (10 out of 19 positions) and XerD (11 out of 19 positions) proteins (Figure 1A). These remarkable sequence similarities suggest that one may expect to identify conserved *dif*-like sequences in these four closely related archaeal genomes based on known properties of bacterial *dif* sites. Finally, XerA proteins are well conserved between these four species (above 85% similarity, Figure S2). *Thermococcales* XerA proteins are thus expected to recognize similar *dif* sites.

We built an algorithm to search for any potential tyrosine recombinase-binding site. We searched for imperfect inverted repeats of 11 to 15 bp separated by spacers ranging from 4 to 10 bp. A total of 481,319 sequences were recovered after this analysis. In order to reduce the sequences to one single most likely *dif* candidate, we selected only sequences that were conserved above 80% similarity in the four genomes. We found six sequences fulfilling this criterion: two were shawn to be spacer sequences of CRISPRs [30], [31], three were imperfect inverted repeats of 11 bp separated by long spacers (one of 8 bp and two of 10 bp), and only one sequence in each genome was composed of 11 bp imperfect inverted repeats separated by a 6 bp spacer (Figure 1B). Strikingly, these sequences are 100% conserved between *P. horikoshii* and *P. furiosus*, have three mismatches with that of *Pyrococcus abyssi* and seven with that of *T. kodakaraensis*. Moreover, these four predicted sites share many positions with the bacterial *dif* consensus sites (Figure 1B). The three *Pyrococcus* sites show 14 out of 28 conserved positions of the bacterial *dif*-consensus, and the *T. kodakaraensis* site shows 18 out of 28 (Figure 1B). The same site search was performed on the *T. gammatolerans*, *T. onnurineus* and *T. sibiricus* genomes and led to the identification of unique sites showing the same level of conservation with the bacterial *dif*-consensus (Figure S3). Further analysis of the *dif* sites environment in *Thermococcales* genomes revealed that all *dif* sites are surrounded by conserved flanking sequences (Figure S3).

**Figure 2:**
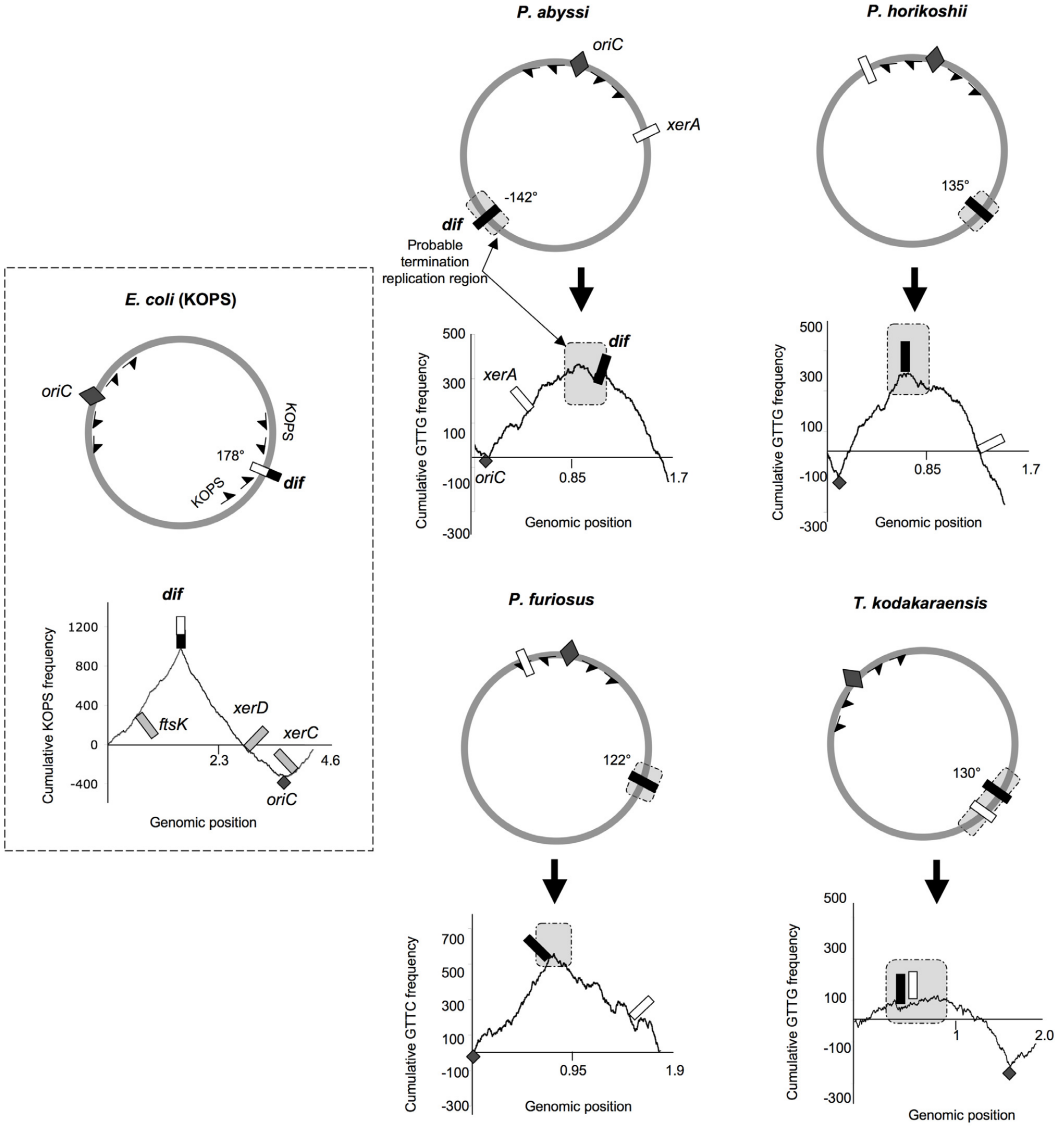
Genomic localization of *dif* sites and *xer* genes. The *E. coli* KOPS cumulative skew produces a pyramidal shaped graphic with the *dif* site (black and white rectangle) located at the summit (178° from *oriC*). *xerC*, *xerD* and *ftsK* genes are located in the *oriC* region. ASPS skew graphics from *Thermococcales* show rounded shapes where *oriC* (dark gray diamond) can be localized precisely and where putative regions of replication termination (light gray rectangles) can be broadly determined. GTTG is the most skewed 4 nt sequence in *P. abyssi*, *P. horikoshii* and *T. kodakaraensis* genomes whereas GTTC is the most skewed 4 nt sequence in *P. furiosus* genome. *Thermococcales dif* sites (black rectangles) are all located at the summit of the ASPS skews graphics, at 120–142° from *oriC*, in putative replication termination regions. The *xerA* gene (white rectangles) positions are more variable. Genomic coordinates of *oriC*, *dif* and *xerA* genes can be found in Table S1.

As in the canonical bacterial model, *Thermococcales dif* sites predicted by our analysis were not located next to the *xerA* genes (Figure 2 and Figure S4). The position of the *xerA* genes relative to the replication origins (*oriC*) was highly variable (Table S1), whereas the predicted *dif* sites were located within the second quarter of the genome for *P. horikoshi*, *P. furiosus* and *T. kodakaraensis* (135°, 122° and 130° from *oriC*, respectively) and in the third quarter (−142° from *oriC*) for *P. abyssi* (Figure 2). In the latter case, the difference in position could be a consequence of the large fragment inversion containing *oriC* that recently occurred in this species [32]. The conservation of *dif* site positions relative to *oriC* in the four *Thermococcales* (between 122° and 142°) is especially striking since these genomes have been extensively rearranged by chromosome recombination, as indicated by the patterns obtained from whole genome alignments (Figure S5).

The *dif*-like sites identified in our analyses do not localize precisely at 180° from *oriC*. However, the predicted *dif* site of *P. abyssi* is located into a late replicating fragment of the genome [33]. *dif* sites positions are therefore compatible with a localization in the terminus region of chromosome replication. It is not known if the two replication forks always meet at the same point in *Thermococcales*. A precise site for the terminus of DNA replication in *Thermococcales* genomes cannot be predicted by using GC skew analysis because, in contrast to the sharp peak observed at *oriC*, the potential termination region appears as a broad distribution [33], [34]. The terminus region appears to be especially prone to chromosomal rearrangement in *Thermococcales* [32], possibly explaining this lack of resolution.

We next extended our analysis to archaeal genomes outside of the *Thermococcales* group. Using the *dif* sites and flanking sequences found in *Thermococcales*, we constructed a Hidden Markov Model with HMMER2 [35] and searched other archaeal genomes for potential *dif* sequences. As an example, a single statistical-significant sequence matching the *Thermococcales dif* sites was found in the *Methanosphaera stadtmanae* genome. This site is located at about 180°C from *oriC* (Figure S6), and is surrounded by imperfect inverted repeats. We then selected three *Sulfolobales* genomes to search for *dif* sites in Crenarchaeota. *Sulfolobus* species possess multiple replication origins [36], [37] raising the possibility that an alternative to the canonical Xer recombination system may occur in these organisms. We used the same initial methodology that was applied to *Thermococcales* genomes. Unique *dif* sites were found for *S. acidocaldarius* and *S. tokodaï*, whereas two copies of this site were found at the same chromosomal location in the *S. solfataricus* genome (Figure S7). As opposed to Euryarchaeota, the predicted *dif* sites localized close to *xerA* genes, and were flanked on only one side by a short conserved sequence of 13 bp.

### Identification of polarized sequences that point to the predicted *dif* sites

In Bacteria, the directionality of FtsK-mediated DNA translocation is determined by octamers that are polarized on each arm of the chromosome with their orientation switching at the *dif* site [16]–[18]. Although Archaea lack an FtsK homologue, we searched for the most frequent and skewed sequences in *Thermococcales* genomes by using the R'MES program ([38], see Materials and Methods). We used the *E. coli* genome to validate this methodology and, as expected, we found that KOPS are the most over-represented skewed 8nt-long sequences. The corresponding diagram shows a sharp peak corresponding to the position of the *E. coli dif* site (Figure 2). We then analyzed all possible sequences of 4 to 8 nucleotide-long in the four *Thermococcales* genomes. We identified GTTG as the most over-represented and skewed sequence in the *P. abyssi*, *P. horikoshi* and *T. kodakaraensis* genomes, and GTTC in the *P. furiosus* genome. For *Thermococcales* genomes, the cumulative frequency of these 4 nucleotide sequences (Archaea Short Polarized Sequences, ASPS) does not give a perfect triangle-shaped diagram as observed for Proteobacteria (Figure 2) or Firmicutes where *dif* sites locate exactly at the KOPS skew inversion. Nevertheless, in the case of the three *Pyrococcus* genomes, the cumulative ASPS skews diagrams displayed a sharp optima precisely located next to the *dif*-like sites identified *in silico* (Figure 2). The optimum was located very close to the *dif* sites in the cases of *P. horikoshi* and *P. furiosus*, whereas it was located more to the left in the case of *P. abyssi* (around 160° instead of 142°). This shift could be due to a recent transposition of two chromosomal segments that occurred in the terminus region of this species [32]. In the case of *T. kodakaraensis*, we only obtained the sharp minimum corresponding to the replication origin, whereas the opposite region appeared as a broad peak containing the predicted *dif* site. The same analysis was extended to other archaeal genomes where *dif* sites were predicted, and revealed that both euryarchaeal and crenarchaeal genomes harbour ASPS (Figure 2; Figures S4, S6, S7). Remarkably, the *M. stadtmanae* ASPS skew displays the triangle-shaped diagram observed in Bacteria, with the predicted *dif* site precisely located at the skew inversion (Figure S6).

### *P. abyssi* XerA protein binds to the predicted *dif* site

**Figure 3:**
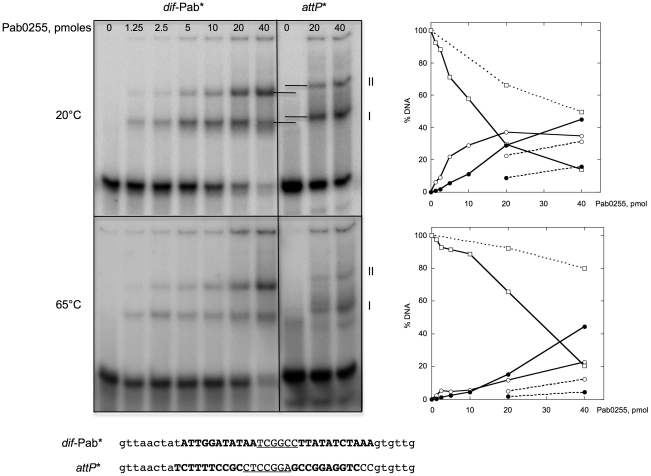
Binding of *P. abyssi* XerA to specific and non-specific DNA substrates. Increasing amounts of XerA were incubated at 20°C or 65°C with two different double-stranded oligonucleotides 5′end-labeled on their top strand. Reactions were loaded onto 5% non denaturing acrylamide gels containing 5% glycerol. Electrophoresis was performed in TGE buffer (50 mM Tris, pH 7.5, 8 mM glycine, 0.1 mM EDTA) at 4°C for 4 h at 7.5 V/cm. The DNA-protein complexes were visualized by phosphore-imaging. Right panel: quantification of free and bound DNA as a function of Pab0255 amount. Plain lines, *dif*-Pab substrate; dotted lines, *att*P substrate. Free DNA, square; complex I, white circle; complex II, black circle.

To test our *in silico* predictions, we purified to homogeneity the *P. abyssi* XerA protein (Pab0255) as a recombinant protein. Recognition of the predicted *dif* site by the *P. abyssi* XerA protein was first evaluated by Electrophoretic Mobility Shift Assay (EMSA). Double stranded oligonucleotides corresponding to the *P. abyssi dif* site were incubated with increasing amounts of *P. abyssi* XerA protein at 20° and 65°C (Figure 3). As a control, *P. abyssi* XerA protein was also incubated in presence of a non-specific DNA site corresponding to the minimal recombination site (*attP*) of another archaeal tyrosine recombinase, the SSV1 integrase [39]. XerC and XerD from *E. coli* have only been shawn to bind to an oligonucleotide containing the *E. coli dif* site [40]. In contrast, *P. abyssi* XerA was able to bind to both substrates, with two protein-DNA complexes detected in each case (Figure 3). However, complex migration was different between the two DNA substrates, with *P. abyssi* XerA/*dif* complexes showing a higher mobility than *P. abyssi* XerA/*attP* complexes. Furthermore, the *P. abyssi* XerA protein presented a preference for the *P. abyssi dif* site as compared to the non-specific substrate, with a 4 fold increase in complex formation at 20°C and an 8 fold increase at 65°C (Figure 3). At the *P. abyssi* optimal growth temperature (90°C), XerA binding to the *dif* site should therefore be highly specific. Competition experiments further confirmed that XerA has a much higher affinity for its *dif* site than for a heterologous tyrosine recombinase binding site (Figure S8).

### *P. abyssi* XerA protein specifically recombines plasmids containing the predicted *dif* site

**Figure 4:**
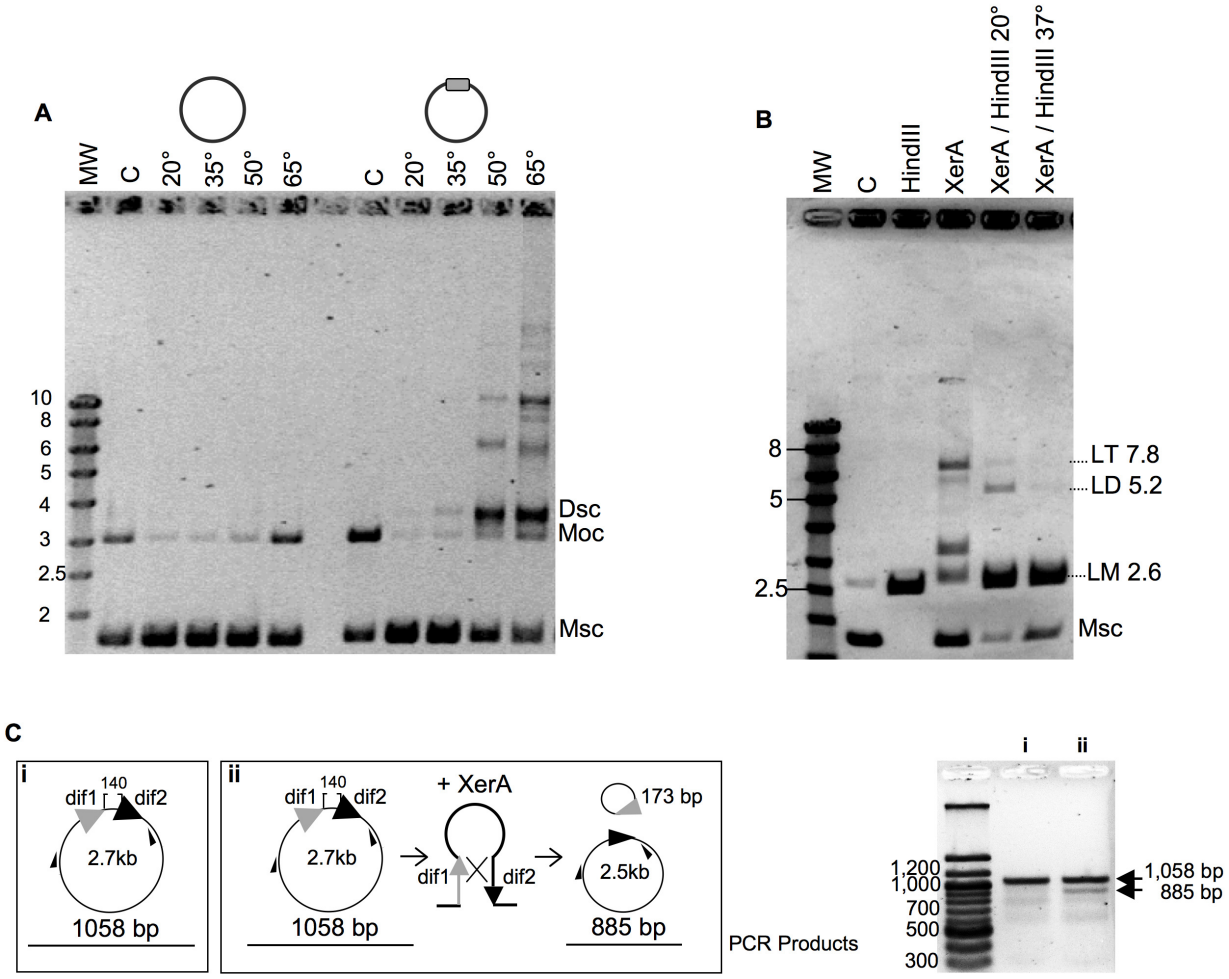
Recombination activity of *P. abyssi* XerA. A. Temperature dependence of the reaction. XerA was incubated at different temperatures with a plasmid containing or not the predicted *dif* site (gray box). MW: molecular weight marker (identified in Kbp on the left). C: control without protein. The control assay was performed at 65°C. Msc: supercoiled monomer. Moc: open circular monomer. Dsc: supercoiled dimer. B. Recombination product analysis. C: control without protein. *Hin*dIII, pBend2*dif* full restriction by *Hin*dIII. XerA: recombination reaction, 1h at 65°C. XerA/*Hin*dIII 20°C: Recombination reaction followed by restriction with one unit of *Hin*dIII, for 1 h at 20°C. XerA/*Hin*dIII 37°C: same as before, but restriction performed at 37°C. Msc: supercoiled monomer. LM: linearized monomer (2.6 kbp). LD: linearized dimer (5.2 kbp). LT: linearized trimer (7.8 kbp). C. Resolution reaction. The substrate used is diagramed in (i) and the product in (ii). Small black arrows correspond to the hybridization position of the oligonucleotides used in the PCR assay. PCR fragments obtained on the substrate (i) or on the reaction products (ii) were analyzed by agarose gel electrophoresis (right).

We next searched for full or partial site-specific recombination activity. In the case of XerCD, *in vitro* recombination at *dif* sites requires the C-terminal domain of FtsK [15]. However, *dif*-dependent DNA relaxation has been observed for XerC and XerD [41]. In order to test for such activity, we cloned an oligonucleotide containing the predicted *dif* site into a plasmid vector. After incubation of XerA with this substrate, reactions products were analyzed by agarose gel electrophoresis. Whereas incubation of the control plasmid (without *dif* site) with *P. abyssi* XerA protein did not reveal any reaction product, addition of the protein to the *dif*-containing plasmid led to the appearance of several new bands of lower mobility than the open circular (Moc) form of the substrate (Figure 4A). The migration of the major product suggested that it may correspond to the supercoiled form of a dimeric plasmid (Dsc), while the other products may correspond to increasing multimers of the *dif*-containing plasmid. The reaction was strongly dependent on temperature (Figure 4A), as expected for a reaction catalyzed by a protein from a hyperthermophilic organism. A single product, migrating slightly above the open circular substrate form appeared when the incubation was performed at 20° or 35°C. The amount of product strongly increased when the incubation was performed at 50° and 65°C, reaching an amount roughly equivalent to that of the remaining monomeric supercoiled (Msc) substrate. Several new products of low mobility were detected when the reaction was performed above 50°C, and their relative amounts increased from 50° to 65°C. To determine whether the reaction products generated by the *P. abyssi* XerA were indeed multimers of the *dif*-containing plasmid, we took advantage of a unique *Hin*dIII restriction site present on the plasmid. We assumed that partial *Hin*dIII digestion of the reaction products would produce linear multimeric plasmids that could be identified by their size. The products of a *P. abyssi* XerA catalyzed reaction performed for 20 minutes at 65°C were incubated with one unit of *Hin*dIII for one hour, either at 37°C for full digestion or at 20°C for partial digestion (Figure 4B). At 37°C, digestion of reaction products produced only linear DNA of the monomeric size (LM, 2.6 kb), indicating that all reaction products were multimers of *dif*-containing plasmids which were cleaved at all available *Hin*dIII sites (Figure 4B). *Hin*dIII digestion at 20°C produced two additional bands of linear DNA with the expected molecular weight for linear dimers (LD, 5.2 kb) or linear trimers (LT, 7.8 kb) of the *dif*-containing plasmid. This result indicates that *P. abyssi* XerA can recombine *dif*-containing plasmids in the absence of protein partners, producing multimeric forms of the initial substrate. The specificity of the reaction was further controlled by using as substrate a plasmid containing the *attP* site (Figure S9). No recombination activity could be detected on this substrate, further indicating that binding of XerA to the *att*P site is non specific.

We followed the time course formation of multimeric plasmids by *P. abyssi* XerA on the *dif*-containing plasmid at 65°C. Plasmid dimers were obtained after five minutes incubation and plasmid multimers were detected after 10 minutes (Figure S9). After 30 minutes reaction time, the relative intensity of all bands remained fairly constant suggesting either that the recombination activity had reached enzymatic equilibrium, or that the protein was rapidly inactivated upon incubation at 65°C. However, pre-incubating the protein alone for up to 40 min at 65°C did not reduce the extent of recombination (Figure S9) thus ruling out protein denaturation during the time course assay. This suggests that at the reaction equilibrium, production of multimers from monomers is equivalent to multimer resolution into monomers.

To further validate the resolution activity of the *P. abyssi* XerA protein, we constructed a substrate with two *dif* sites in direct repeat (Figure 4C). The reaction products were analyzed by PCR as both integration and resolution events can occur on this substrate. Resolution events reduced the distance between the two primers from 1058 bp to 885 bp (Figure 4C). Even though integration events were still favoured, as attested by the appearance of plasmid multimers (not shown), a PCR product with a size around 900 bp was detected (Figure 4C). This product indicates that XerA was able to assemble a recombination proficient synaptic complex and that although at low level resolution events also occurred. The *P. abyssi* XerA protein is therefore able to catalyse both resolution and integration depending on the substrate provided in the reaction.

## Discussion

We have shown that the *P. abyssi* XerA protein (homologous to the bacterial XerCD recombinases) specifically recombines a plasmid containing a predicted *dif* sequence present in the *P. abyssi* genome. This *dif* sequence was identified *in silico*, taking into account known features of tyrosine recombinase recombination sites and searching for sequences present in the genomes of four closely related *Thermococcales*. Importantly, whereas the location of *xerA* genes relative to the replication origin (*oriC*) varies from one genome to the other, the positions of the *dif* sites with respect to *oriC* is relatively conserved in all four genomes. These observations strongly suggest that archaeal XerA proteins may be involved in the resolution of chromosome dimers at the terminus of replication.

Interestingly, although Archaea lack a FtsK homologue, we could identify polarized sequences of four nucleotides (ASPS) that define the replication termination region and point towards the predicted *dif* sites in Euryarchaeota. The ASPS are shorter than the KOPS used by FtsK in Bacteria. Strikingly, in most genomes the predicted *dif* site localized at the summit of curves obtained by ASPS cumulative skew analyses. Archaea may therefore use a functional analogue of the bacterial FtsK-KOPS mechanism to produce a *dif*-synaptic complex *in vivo*. It was suggested that the archaeal bipolar DNA helicase HerA, which is probably involved in the processing of double-strand breaks for homologous recombination [42]–[44] may also be a functional analogue of FtsK in Archaea on the basis of their common ATPase domain used for DNA translocation [45]. Even though our results show that *P. abyssi* XerA does not require an accessory protein to catalyse recombination *in vitro*, as opposed to bacterial XerCD which only recombine *dif* sites in the presence of FtsK [12], [15], they do not exclude that a protein partner could either regulate synaptic complex assembly or XerA activity *in vivo*. Indeed, in *Thermococcales* genomes, *dif* sites are flanked by inverted repeats (Figure S4) that may be binding sites for such partner.

Using different *in silico* methodologies, we were able to predict *dif* sequences in other euryarchaeal genomes and crenarchaeal genomes that harbour *xerA* genes. Archaea lacking a *xerA* gene, such as Thaumarchaea and *Pyrobaculum* species, may have recruited another tyrosine recombinase, as happened in some bacterial groups [5], [22].

Although the archaeal and bacterial Xer/*dif* systems use similar *dif* sites, they differ in terms of biochemical properties and reaction mechanisms. Whereas *E. coli* and *B. subtilis* XerCD do not bind a DNA fragment without *dif* site [8], the *P. abyssi* XerA protein can bind with a lower affinity to a heterologous tyrosine recombinase site. However this site is not recognized as a recombination substrate. More significantly, the *P. abyssi* XerA protein can recombine plasmids carrying the *dif* sequence in the absence of protein partner. In contrast, the XerCD activity depends *in vitro* and *in vivo* on FtsK to recombine the chromosomal *dif* site or on other partners (PepA and ArcA or ArgR) to recombine the plasmidic recombination sites *psi* on pSC101 or *cer* on ColE1 [46]–[49]. The ability of *P. abyssi* XerA to recombine *in vitro* a *dif*-containing plasmid without any accessory protein suggests that XerA may also work alone *in vivo*. However we cannot rule out that a protein partner may bind the conserved *dif*-flanking sequences. Such a partner may either control the directionality of the reaction towards resolution or coordinate the recombination activity with the progression of the cell cycle. Alternatively, XerA activity may be limited to replication termination and chromosome segregation by regulating XerA expression at the transcriptional level. In agreement with this view, the *xerA* gene of the crenarchaeon *Sulfolobus acidocaldarius* (Saci 1490) is induced during the G1/S phase and reaches its maximal expression level in the G2 phase of the cell cycle [50].

**Figure 5:**
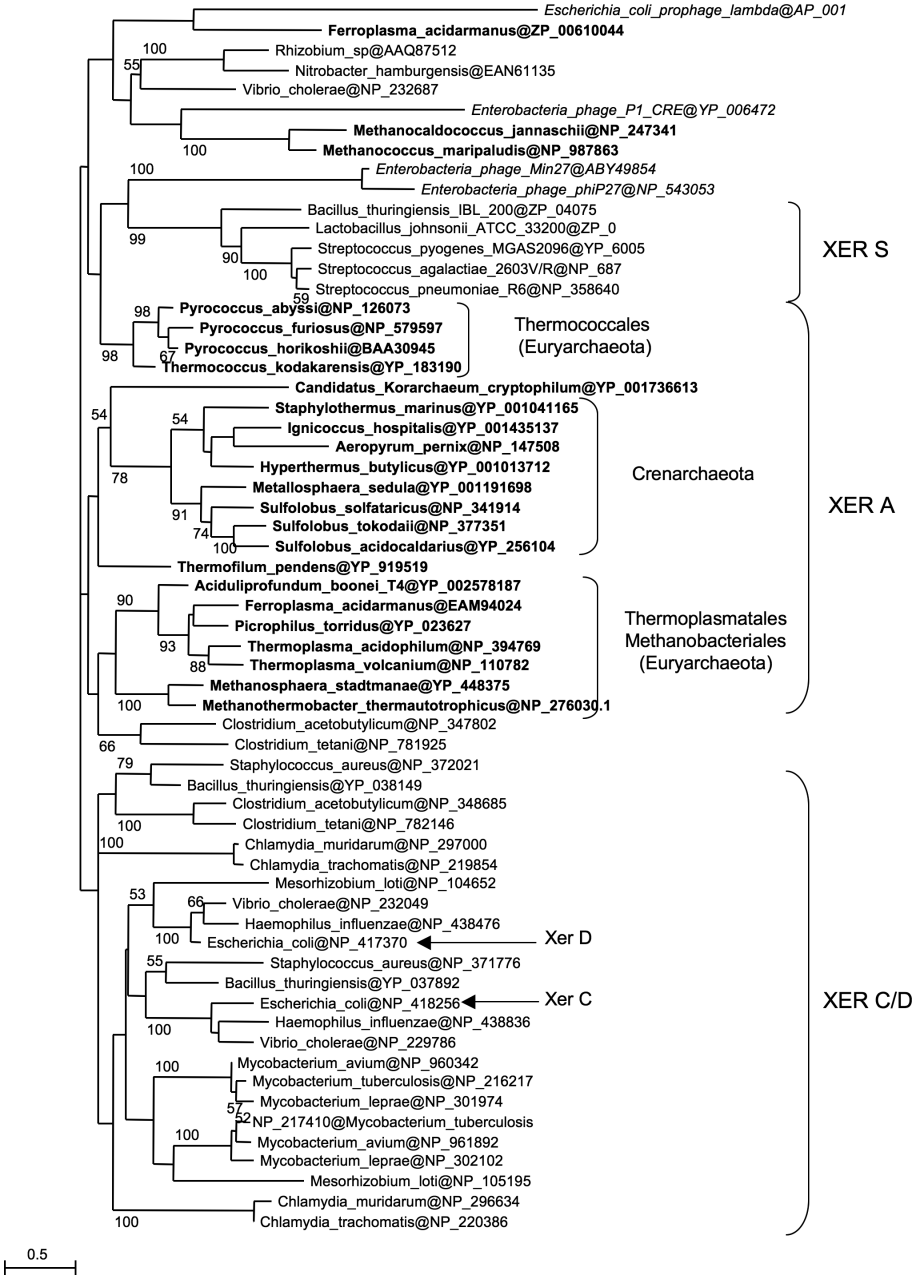
Xer phylogeny. Unrooted maximum likelihood tree of a selection of bacterial and archaeal (bold) Xer homologues and viral tyrosine recombinases (italics). Sequences are identified by their NCBI Reference Sequence. Numbers at nodes represent bootstrap values. The scale bar represents the average number of substitutions per site.

Our results show that Archaea possess a Xer/*dif* system similar to its bacterial counterpart that is likely involved in chromosome dimer resolution. However, the archaeal Xer system displays differences, such as the involvement of a unique protein and the ability to perform site-specific recombination *in vitro* in the absence of accessory proteins. To get a better view of the evolution of the Xer system, we performed a phylogenetic analysis including bacterial and archaeal Xer proteins and a subset of bacteriophage encoded tyrosine recombinases (Figure 5). Unfortunately, the resulting tree is not resolved at most basal nodes, preventing a clear view of the evolutionary relationships of these proteins. However, it shows that bacterial and archaeal homologues are not intermixed, indicating that no recent horizontal gene transfer has occurred between domains. Our data thus suggest that a Xer/*dif* system was present in the common ancestor of Archaea and Bacteria, suggesting that this ancestor had a circular double-stranded DNA genome. However, this raises further issues such as why the replication machinery of Archaea and Bacteria are now composed of non homologous proteins [51]. Alternatively, homologous viral tyrosine recombinases may have been recruited independently in Archaea and Bacteria to be used as Xer/*dif* systems after transition from RNA to DNA genomes [52]. In any case, the presence of a shared Xer-*dif* system in Bacteria and Archaea illustrates the complex origin of modern DNA genomes. Further studies of Xer-*dif* systems in different archaeal and bacterial groups will be now necessary to test alternative scenarios for the origin and evolution of Xer proteins.

## Materials and Methods

### Identification of *dif* candidates

A model searched for all inverted repeats (11 to 15bp) separated by a spacer (4 to 10bp) present in non-coding genomic regions. A consensus sequence was deduced from the alignment of predicted *dif* sites and represented as a sequence logo [53].

### Skew analyses

Statistical significant skewed words in the four *Thermococcales* genomes were determined using the R'MES program (http://mig.jouy.inra.fr/logiciels/rmes/) [38]. Since R'MES reads only one single strand of DNA at time, we artificially defined several ends of replication, starting at 120°, and then moving by 5° steps up to 200° from *oriC*. From these artificially-defined ends of replication to *oriC* we reverse complemented the genomic sequence. The most skewed word for each analyzed genome was selected by comparing the R'MES results. Cumulative skews were calculated using the formula:
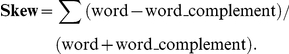



### Genome alignments

Genomes of the four *Thermococcales* where aligned by dot-plot analyses based on BlastP searches (e-value of 1×10^−10^).

### Homology searches and phylogenetic analysis

Xer homologues were searched by BLASTP at the NCBI (http://www.ncbi.nlm.nih.gov/) against complete sequenced archaeal genomes using *E. coli* XerC and XerD proteins as query (threshold of 1×e^−10^). The retrieved homologues were aligned using Muscle and a specific HMM profile was calculated for exhaustive detection of homologues. Using the HMMER program (http://hmmer.janelia.org/) we performed iterative searches until no new homologues were detected in the archaeal genomes. From the resulting dataset, a preliminary phylogenetic analysis allowed to select 62 representative XerC and XerD sequences from several bacterial species and several tyrosine recombinases from plasmids/viruses or from mobile elements integrated into cellular genomes. The final alignment was trimmed to remove ambiguously aligned positions, leading to 222 conserved residues for phylogenetic analysis. A maximum likelihood tree was obtained by using PHYML [54], with the WAG evolutionary model including correction for heterogeneity of evolutionary rates (4 categories+invariant) and statistical support at nodes was calculated by non parametric bootstrap on 100 resampling of the original dataset by PHYML.

### Cloning, expression, and purification of Pab0255

The Pab0255 gene was amplified by PCR using *P. abyssi* genomic DNA as a template, Phusion high-fidelity DNA polymerase (Finnzyme) and the following primers:

5′-GGGAACATATGCACCATCACCATCACCATGAGGAGAGGGAGGAGAGAGTGAGGGATGATACAATTG-3′

5′-TTTTTGCGGCCGCTTAGGAACCCCCGATG-3′

The forward primer allows the addition of six histidine codons in frame with the ATG start codon (underlined). The PCR product was digested by *Nde*I and *Not*I and cloned into a derivative of a pET vector (Novagen). The resulting recombinant plasmid was sequenced prior to being transformed into the *E. coli* expression strain Rosetta(DE3)pLys (Novagen).

Cells were grown in 2xYT medium (BIO101Inc.) at 37°C to A_600nm_ = 1 and expression of Pab0255 was induced by the addition of 0.5 mM IPTG (final concentration). Four hours after induction, cells were harvested by centrifugation, and the pellets resuspended in 40 ml of 50 mM Tris-HCl, pH 8.0, 1 M NaCl and 5 mM β-mercaptoethanol and stored at −20°C. Cells were lysed by sonication and centrifuged at 13, 000× g for 30 min at 25°C. The supernatant was collected and heated for 15 minutes at 70°C. After centrifugation at 13,000× g, the supernatant was loaded onto a Ni^2+^ affinity column (Ni-NTA agarose, Qiagen) pre-equilibrated in the same buffer. The His-tagged Pab0255 was eluted at 20 mM imidazole, and loaded onto a 2 ml HiTrap Heparin (Amersham Biosciences) column pre-equilibrated in a buffer containing 50 mM Tris-HCl pH 7.0, 200 mM NaCl, 1 mM DTT. A NaCl linear gradient (200 mM to 2 M) was developed, and the protein eluted at about 800 mM NaCl. Finally, the protein was loaded onto a cation-exchange SP Sepharose column (Amersham Biosciences) pre-equilibrated in the same buffer, and eluted by a NaCl linear gradient. The purified protein was dialysed against 50 mM Tris pH7.0, 300 mM NaCl, 50% glycerol prior to being stored at −20°C.

### Substrate preparation

The following 43 nt long oligonucleotides containing the top and bottom strands of the predicted *dif* site from *P. abyssi* (Pab) or minimal *attP* site from SSV1 [39] were purchased from Eurogentec:

Pab-Dif-Top 5′-gttaactatATTGGATATAATCGGCCTTATATCTAAAgtgttg-3′

Pab-Dif-Bottom 5′-caacacTTTAGATATAAGGCCGATTATATCCAATatagttaac-3′

attP-Top 5′-gttaactaTCTTTTCCGCCTCCGGAGCCGGAGGTCCCgtgttg-3′

attP-Bottom 5′-caacacGGGACCTCCGGCTCCGGAGGCGGAAAAGAtagttaac-3′

For binding assays, top strand oligonucleotides were 5′-end labeled by using [g-^32^P]ATP and T_4_ polynucleotide kinase. Unincorporated nucleotides were removed by spin dialysis, and the labeled oligonucleotide was then hybridized with a 2-fold excess of unlabeled complementary strand in TE buffer (10 mM Tris, pH 8.0, 1 mM EDTA).

### Electrophoretic mobility shift assays

The DNA binding reactions were carried out in 20 µl of a mixture composed of 0.5 µM 5′-end labeled *dif* substrate or *attP* substrate, increasing amounts of Pab0255 in a binding buffer composed of 50 mM Tris pH 7.5, 30 mM NaCl and 0.5 µg poly(dIdC).poly(dIdC). Incubation was performed for 30 min at either 20°C or 65°C, and then 5 µl of 5× loading buffer (10 mM Tris pH 7.5, 1 mM EDTA, 20% glycerol, 0.1 mg/ml BSA, 0.1% xylene cyanol) was added to the binding reactions. The samples were loaded onto 8% polyacrylamide gels (30∶0.5 acrylamide∶bisacrylamide), and electrophoresis performed in 1× TGE buffer (50 mM Tris, 8 mM Glycine, 0.1 mM EDTA) at 4°C for 4 h at 7 V/cm. The DNA-protein complexes were visualized by autoradiography and phosphorimaging.

### Recombination assays

The 43 bp double stranded oligonucleotide containing the predicted *P. abyssi dif* and the 43 bp double stranded oligonucleotide containing the *att*P site were cloned into the pBend2 (2.6 Kbp) vector [55]. pBend2, pBend2-*dif* and pBend2-*att*P plasmids were purified on CsCl gradients. Recombination reactions were performed in 20 µl of reaction mixture consisting of 30 mM Tris pH 7.5, 50 µg/ml bovine serum albumin, 50 mM NaCl, 500 ng of plasmid and 40 pmol of XerA protein. The reaction mixture was incubated at 65°C (unless otherwise stated) and at the times indicated, quenched with SDS (0.5% final) and 10× loading buffer (100 mM EDTA, 5% SDS, 40% glycerol, 0.35% Bromphenol blue) was added. Reaction mixes were loaded on a 1.2% agarose gel, and electrophoresis performed in 1× TAE buffer at room temperature for 3 hr at 4 V/cm with buffer circulation. DNA was visualized by staining with ethidium bromide.

## Supporting Information

Figure S1 Alignment of the C-terminal domain of Xer proteins from the XerD, XerC, XerA and XerS subfamilies. Left panel: *dif* binding motif alignment. The XerA putative *dif* binding motif show high residues conservation with both XerC and XerD motifs. *Thermococcales* XerA harbour the XerC ‘XRX’ motif signature. XerS proteins show very few residues conserved, meaning that they belong to other tyrosine recombinase subfamily. Right panel: catalytic domain of tyrosine recombinases. Catalytic residues are highlighted in white bold lettering. Note that two catalytic residues apart from the highly conserved motif are not represented here.
(0.09 MB PDF)

Figure S2 Xer similarity scores. A. Top five *E. coli* XerC and XerD matches in complete sequenced archaeal genomes. B. Similarities between XerA from *Thermococcales*.
(0.06 MB PDF)

Figure S3 *Thermococcales* predicted *dif* sites and conserved flanking regions. Alignment of predicted *dif* sites and conserved flanking sequences. The flanking sequences are approximately 23 bp long and AT rich. A consensus sequence was deduced and is represented as a sequence logo (see [53] in the main text).
(0.08 MB PDF)

Figure S4 Genomic localization of *dif* sites and *xer* genes. ASPS skew graphics from *T. sibiricus*, *T. onnurineus* and *T. gammatolerans*. TGGT is the most skewed sequence (ASPS) for all species. Symbols are as in Figure 2. Genomic coordinates of *oriC*, *dif* and *xer*A genes can be found in Table S1.
(0.11 MB PDF)

Figure S5 Whole genome alignments of the four *Thermococcales* genomes. A. Alignment of *P. horikoshii* (X-axis) and *P. abyssi* (Y-axis) genomes shows that they share several regions with conserved gene order. *dif* sites (circle) are located in a relatively well-conserved region at 135° from *oriC* (triangle) in *P. horikoshii* and at 142° from *oriC* in *P. abyssi*. The *xerA* gene (square) genomic position is indicated. Regions where replication may end are indicated by dark rectangles on the axes and delimited by doted lines. B,C. Alignments of *P. furiosus* and *P. abyssi* genomes (B) and of *T. kodakaraensis* and *P. abyssi* genomes (C) reveal an extensive gene order loss. However *dif* relative positions with respect to *oriC* are maintained in all these genomes (respectively 122° and 130° from *oriC* in the *P. furiosus* and *T. kodakaraensis* genomes).
(0.31 MB PDF)

Figure S6 Identification and localization of the *M. stadtmanae dif* site. A single statistical-significant sequence matching the *Thermococcales dif* sites was found in *M. stadtmanae* by using HMM search. The *dif* candidate localizes at the ASPS skew inversion.
(0.09 MB PDF)

Figure S7 *Sulfolobales dif* sites. By using the methodology described in the main text of this article on *S. solfataricus*, *S. acidocaldarius* and *S. tokodaii* genomes, one single sequence that fits all of the requirements (two inverted repeats separated by a spacer of 4–8 base pairs, highly conserved between the three genomes and located inside intergenic regions) was found. This potential *dif* candidate is present only once in *S. acidocaldarius* and *S. tokodaii*, but has two copies (only one highly conserved), at the same chromosomal location in *S. solfataricus* genome.
(0.10 MB PDF)

Figure S8 Binding specificity of *P. abyssi* XerA to specific and non-specific DNA substrates. 40 pmoles of XerA were incubated with *dif*-Pab or *att*P substrates at 20°C with increasing amounts of non specific competitor poly(dIdC)_2_. Bottom panel: quantification of free and bound DNA as a function of poly(dIdC)_2_ amount. Plain lines, *dif*-Pab substrate; dotted lines, *att*P substrate. Free DNA, square; complex I, white circle; complex II, black circle.
(0.14 MB PDF)

Figure S9 XerA enzymatic properties. A. XerA substrate specificity. The three substrates were incubated for 1 hr at 65°C with or without 10 pmol of XerA. Recombination products are only observed on the pBend2-*dif* substrate. B: time course of XerA-mediated recombination at 65°C. C: XerA was pre-incubated at different times at 65°C and then mixed with the *dif*-containing plasmid for one hour at 65°C. No difference in activity is observed between the different lanes, indicating that XerA is stable for more than one hour at 65°C.
(0.25 MB PDF)

Table S1 Specific genomic positions of *oriC*-*cdc*6, *xerA* genes and *dif* sites in *Thermococcales* genomes.
(0.06 MB PDF)
