# Supplementary figures and images for: Evidence for a Xer/dif System for Chromosome Resolution in Archaea
Source: PLoS Genet. 2010 Oct 21;6(10):e1001166. doi: 10.1371/journal.pgen.1001166 (PMC2958812; doi:10.1371/journal.pgen.1001166)

Figure S1

XER ALIGNMENT

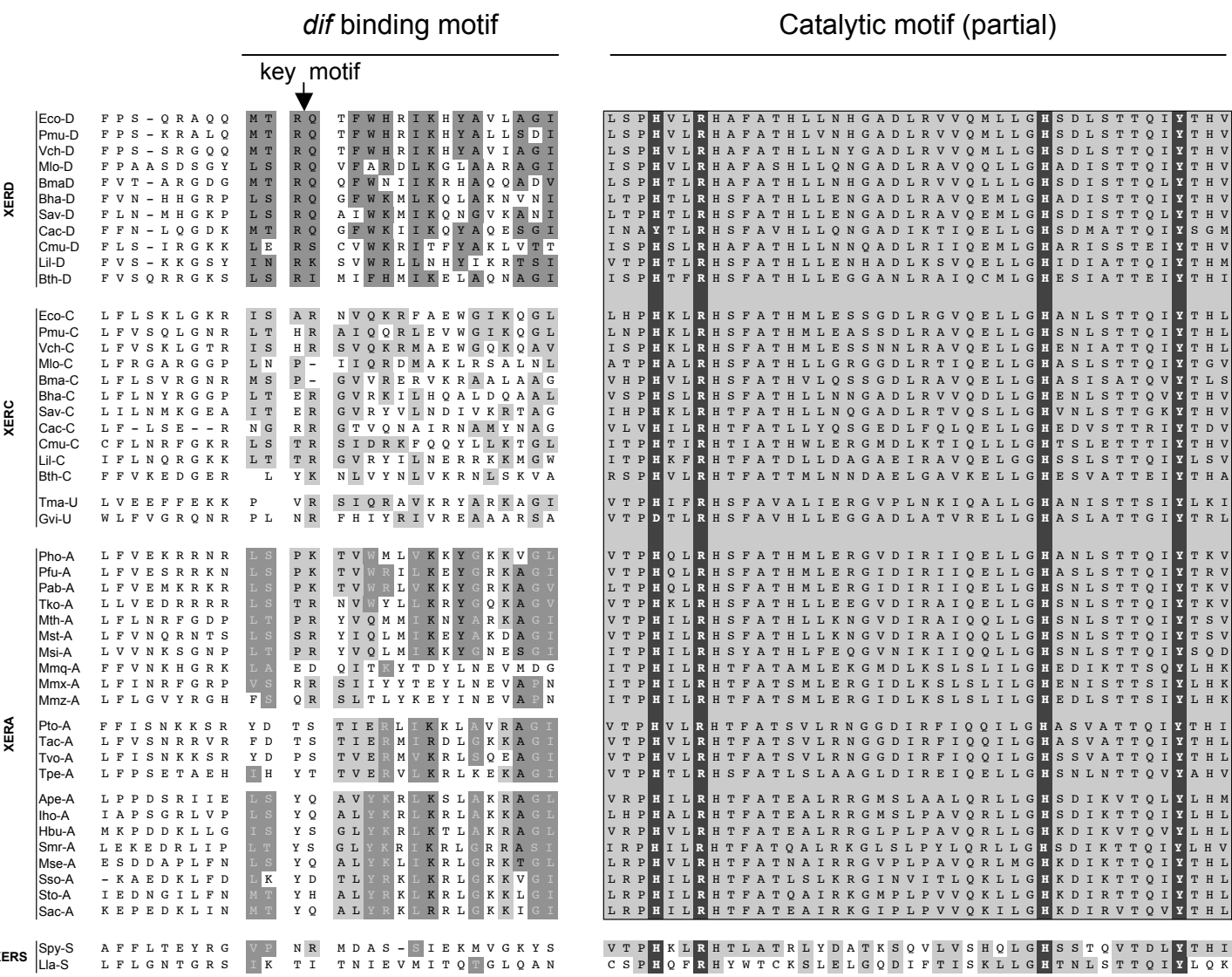

Supplement: Figure S1 — Alignment of the C-terminal domain of Xer proteins from the XerD, XerC, XerA and XerS subfamilies. Left panel: dif binding motif alignment. The XerA putative dif binding motif show high residues conservation with both XerC and XerD motifs. Thermococcales XerA harbour the XerC ‘XRX’ motif signature. XerS proteins show very few residues conserved, meaning that they belong to other tyrosine recombinase subfamily. Right panel: catalytic domain of tyrosine recombinases. Catalytic residues are highlighted in white bold lettering. Note that two catalytic residues apart from the highly conserved motif are not represented here. (0.09 MB PDF) [file pgen.1001166.s001.pdf]

## Consensus

## Consensus

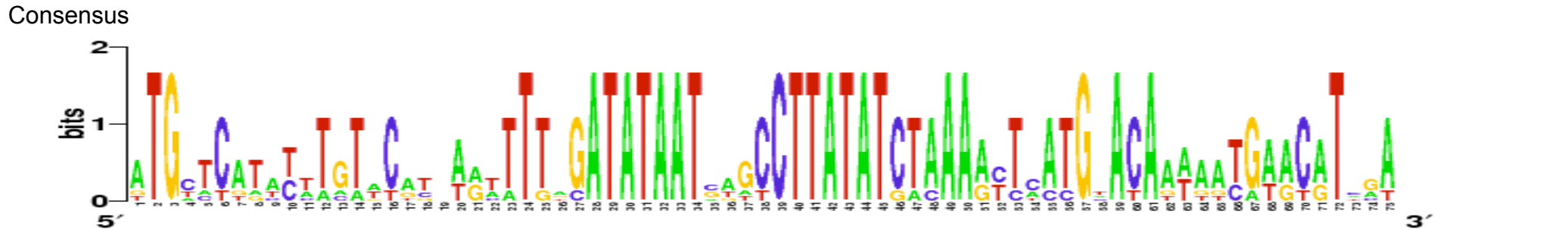

Supplement: Figure S3 — Thermococcales predicted dif sites and conserved flanking regions. Alignment of predicted dif sites and conserved flanking sequences. The flanking sequences are approximately 23 bp long and AT rich. A consensus sequence was deduced and is represented as a sequence logo (see [53] in the main text). (0.08 MB PDF) [file pgen.1001166.s003.pdf]

Figure S5

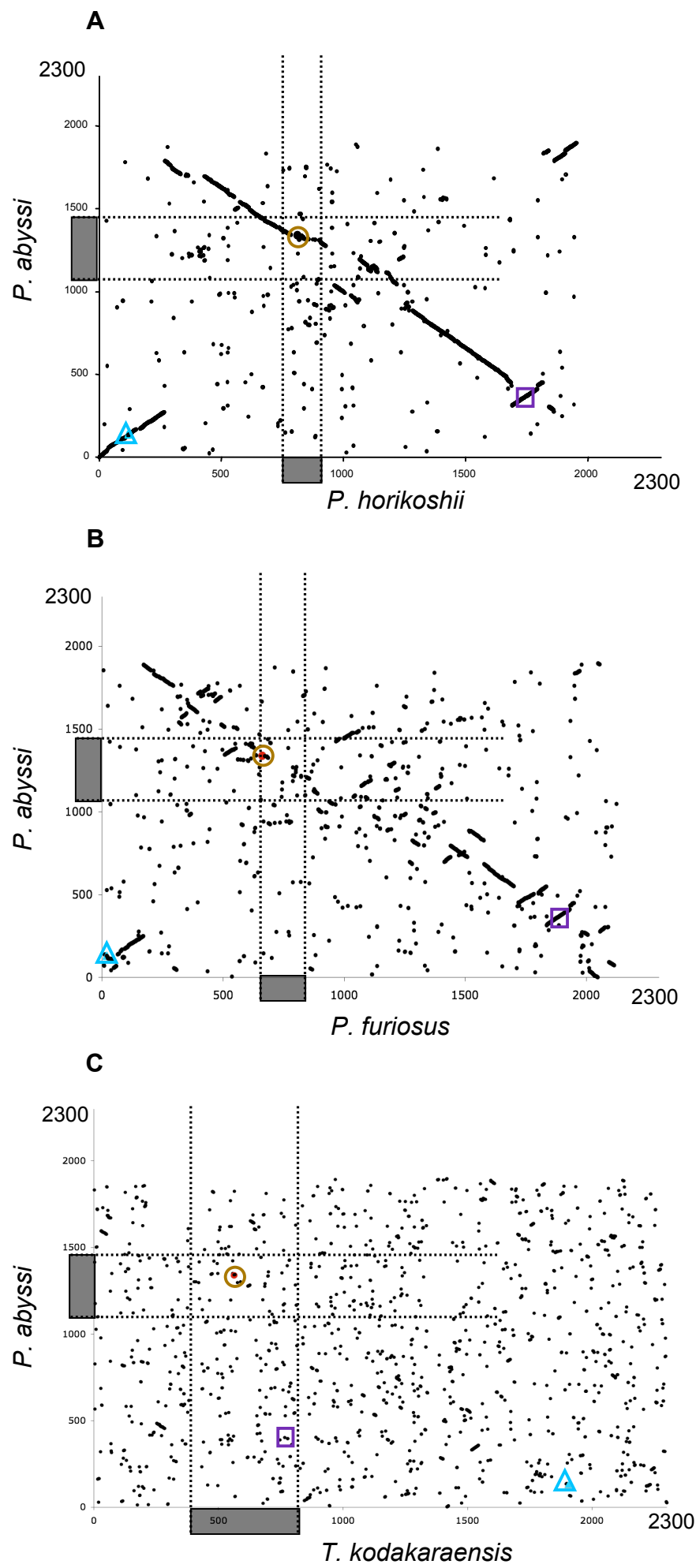

Supplement: Figure S5 — Whole genome alignments of the four Thermococcales genomes. A. Alignment of P. horikoshii (X-axis) and P. abyssi (Y-axis) genomes shows that they share several regions with conserved gene order. dif sites (circle) are located in a relatively well-conserved region at 135° from oriC (triangle) in P. horikoshii and at 142° from oriC in P. abyssi. The xerA gene (square) genomic position is indicated. Regions where replication may end are indicated by dark rectangles on the axes and delimited by doted lines. B,C. Alignments of P. furiosus and P. abyssi genomes (B) and of T. kodakaraensis and P. abyssi genomes (C) reveal an extensive gene order loss. However dif relative positions with respect to oriC are maintained in all these genomes (respectively 122° and 130° from oriC in the P. furiosus and T. kodakaraensis genomes). (0.31 MB PDF) [file pgen.1001166.s005.pdf]

Figure S8

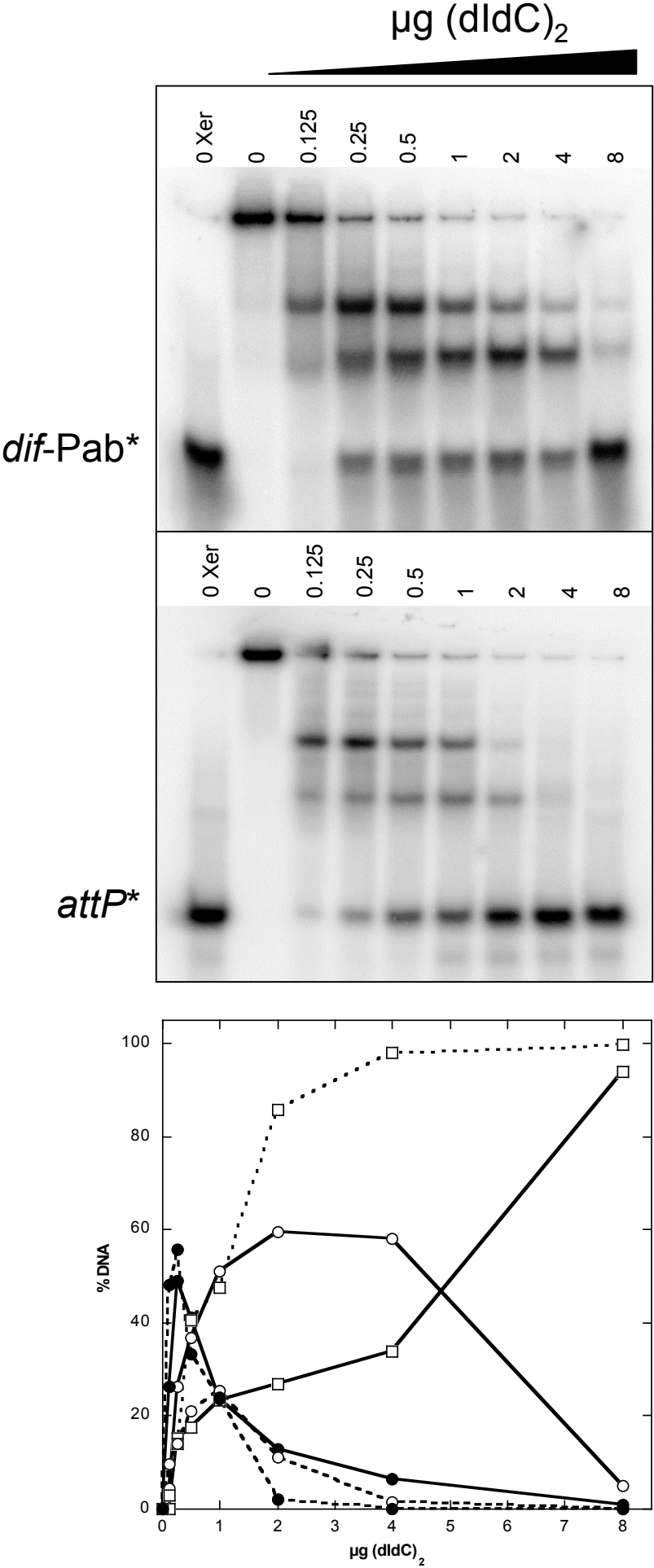

Supplement: Figure S8 — Binding specificity of P. abyssi XerA to specific and non-specific DNA substrates. 40 pmoles of XerA were incubated with dif-Pab or attP substrates at 20°C with increasing amounts of non specific competitor poly(dIdC)2. Bottom panel: quantification of free and bound DNA as a function of poly(dIdC)2 amount. Plain lines, dif-Pab substrate; dotted lines, attP substrate. Free DNA, square; complex I, white circle; complex II, black circle. (0.14 MB PDF) [file pgen.1001166.s008.pdf]

Figure S9

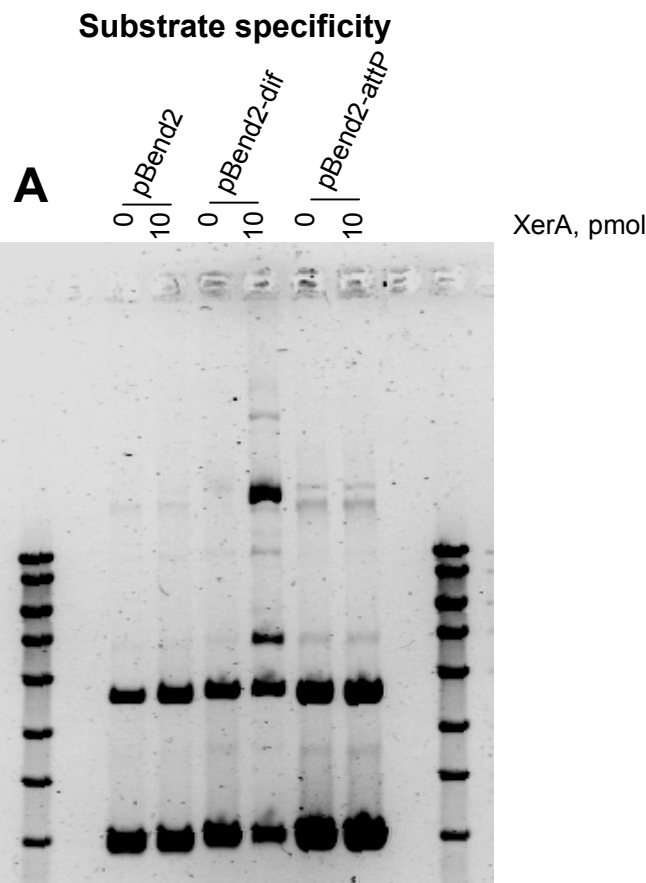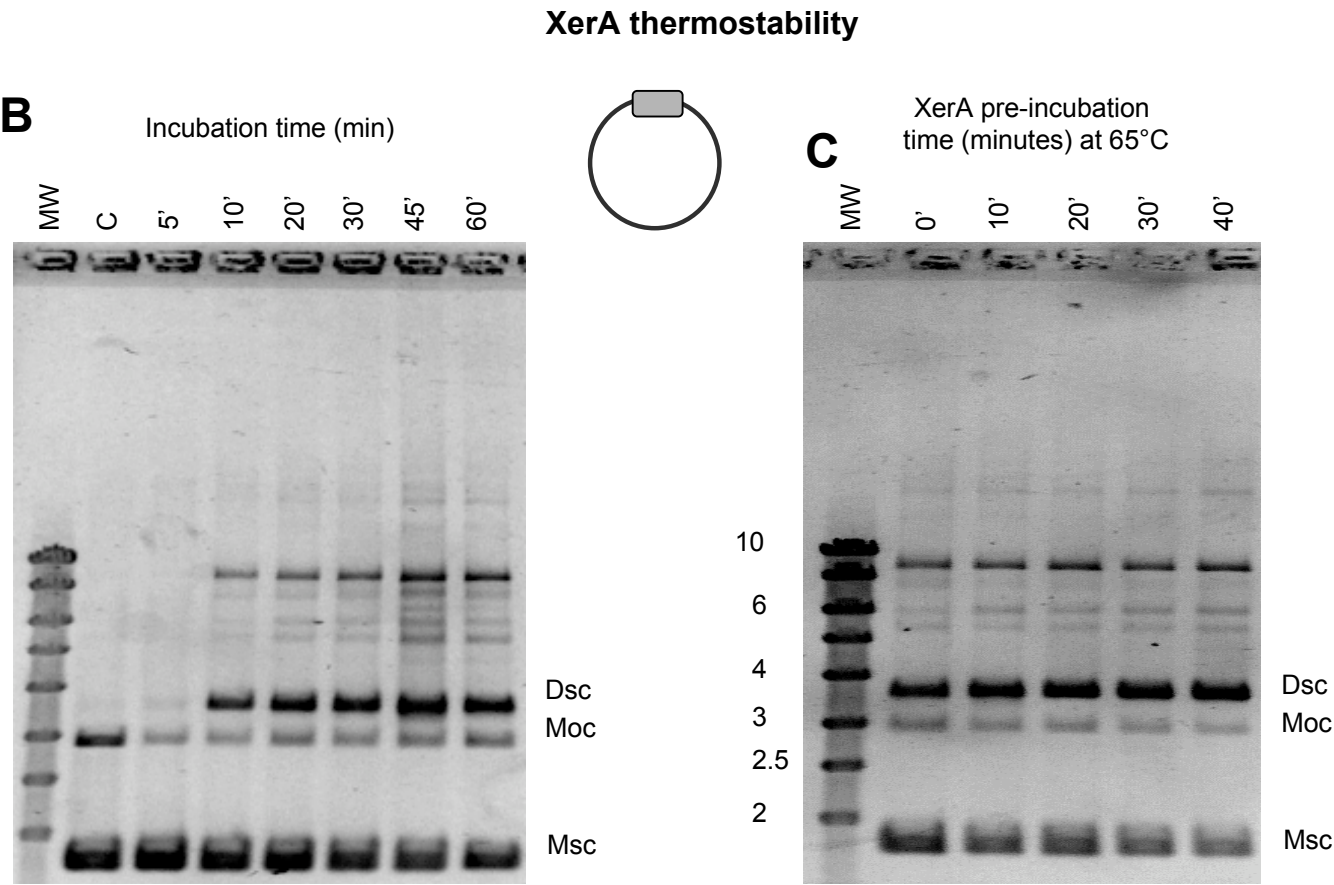

Supplement: Figure S9 — XerA enzymatic properties. A. XerA substrate specificity. The three substrates were incubated for 1 hr at 65°C with or without 10 pmol of XerA. Recombination products are only observed on the pBend2-dif substrate. B: time course of XerA-mediated recombination at 65°C. C: XerA was pre-incubated at different times at 65°C and then mixed with the dif-containing plasmid for one hour at 65°C. No difference in activity is observed between the different lanes, indicating that XerA is stable for more than one hour at 65°C. (0.25 MB PDF) [file pgen.1001166.s009.pdf]
