# Supplementary material for: Evidence for a Xer/dif System for Chromosome Resolution in Archaea
Source: PLoS Genet. 2010 Oct 21;6(10):e1001166. doi: 10.1371/journal.pgen.1001166 (PMC2958812; doi:10.1371/journal.pgen.1001166)
Supplement: Figure S2 — Xer similarity scores. A. Top five E. coli XerC and XerD matches in complete sequenced archaeal genomes. B. Similarities between XerA from Thermococcales. (0.06 MB PDF) [file pgen.1001166.s002.pdf]

Figure S2

A

Top XerC matches in Archaea

	length	identity	SW-score	bits
P. horikoshii	285	0.398	485	116
P. abyssi	286	0.394	481	115
P. furiosus	286	0.377	472	113
T. kodakaraensis	282	0.357	501	120
M. thermoautotrophicum	311	0.336	482	116

Top XerD matches in Archaea

	length	identity	SW-score	bits
P. abyssi	286	0.371	527	126
T. kodakaraensis	282	0.359	490	118
P. horikoshii	285	0.352	494	118
M. thermoautotrophicum	295	0.350	489	117
P. furiosus	286	0.350	484	116

B

XerA similarities among *Thermococcales*

	P. abyssi	***P. horikoshii***	***P. furiosus***	***T. kodakaraensis***
P. abyssi	100%			
P. horikoshii	93%	100%		
P. furiosus	89%	89%	100%	
T. kodakaraensis	85%	86%	85%	100%
