# Supplementary material for: Evidence for a Xer/dif System for Chromosome Resolution in Archaea
Source: PLoS Genet. 2010 Oct 21;6(10):e1001166. doi: 10.1371/journal.pgen.1001166 (PMC2958812; doi:10.1371/journal.pgen.1001166)
Supplement: Figure S4 — Genomic localization of dif sites and xer genes. ASPS skew graphics from T. sibiricus, T. onnurineus and T. gammatolerans. TGGT is the most skewed sequence (ASPS) for all species. Symbols are as in Figure 2. Genomic coordinates of oriC, dif and xerA genes can be found in Table S1. (0.11 MB PDF) [file pgen.1001166.s004.pdf]

Figure S4

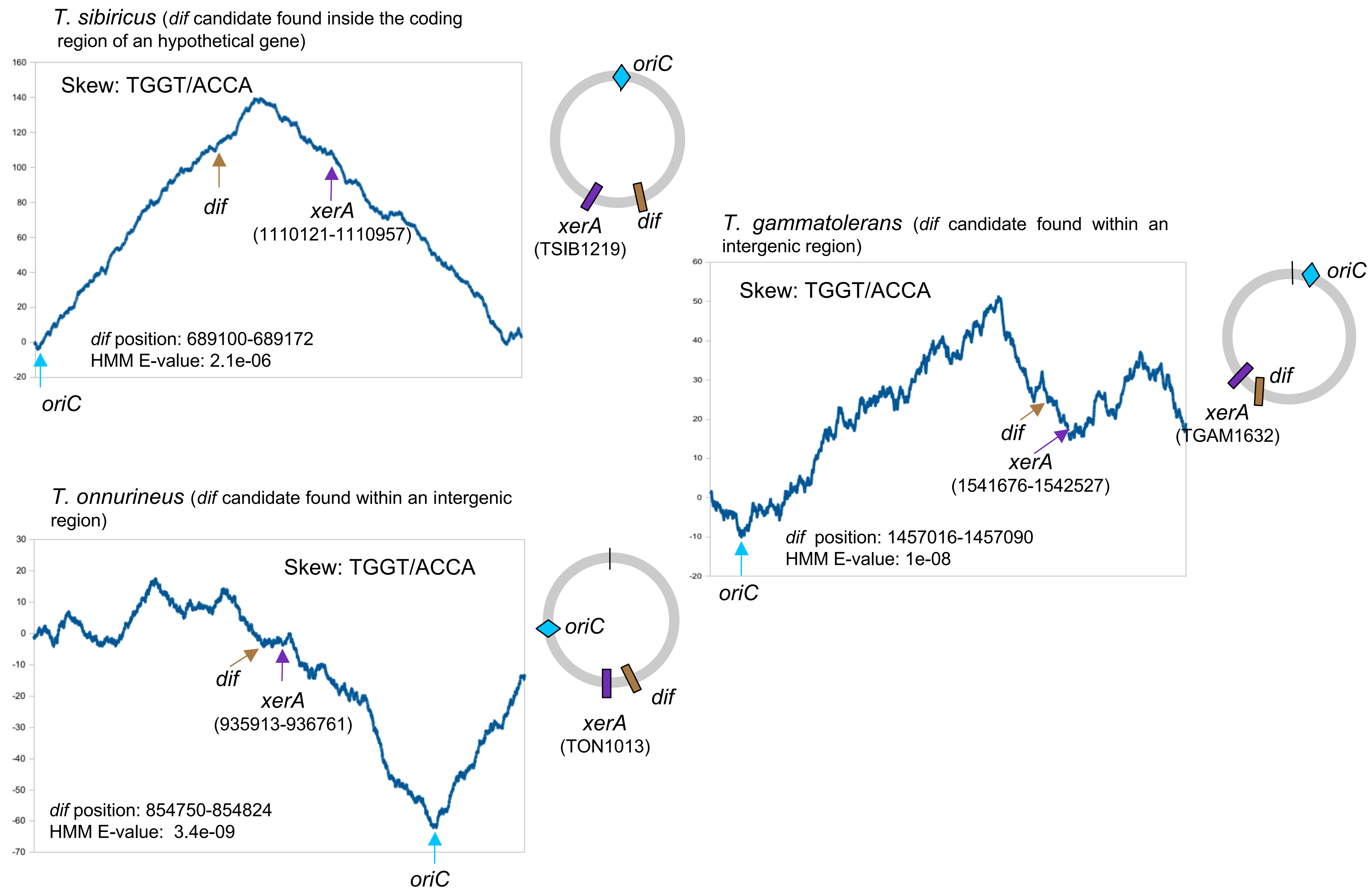

T. sibiricus (dif candidate found inside the coding region of an hypothetical gene)
Skew: TGGT/ACCA
dif
xerA
(1110121-1110957)
oriC
dif position: 689100-689172
HMM E-value: 2.1e-06
oriC
xerA
(TSIB1219)
dif
T. gammatolerans (dif candidate found within an intergenic region)
Skew: TGGT/ACCA
dif
xerA
(1541676-1542527)
dif position: 1457016-1457090
HMM E-value: 1e-08
oriC
oriC
dif
xerA
(TGAM1632)
T. onnurineus (dif candidate found within an intergenic region)
Skew: TGGT/ACCA
dif
xerA
(935913-936761)
dif position: 854750-854824
HMM E-value: 3.4e-09
oriC
oriC
dif
xerA
(TON1013)
