# Supplementary material for: Evidence for a Xer/dif System for Chromosome Resolution in Archaea
Source: PLoS Genet. 2010 Oct 21;6(10):e1001166. doi: 10.1371/journal.pgen.1001166 (PMC2958812; doi:10.1371/journal.pgen.1001166)
Supplement: Figure S6 — Identification and localization of the M. stadtmanae dif site. A single statistical-significant sequence matching the Thermococcales dif sites was found in M. stadtmanae by using HMM search. The dif candidate localizes at the ASPS skew inversion. (0.09 MB PDF) [file pgen.1001166.s006.pdf]

Figure S6

M. stadtmanae (*dif* candidate found within an intergenic region)

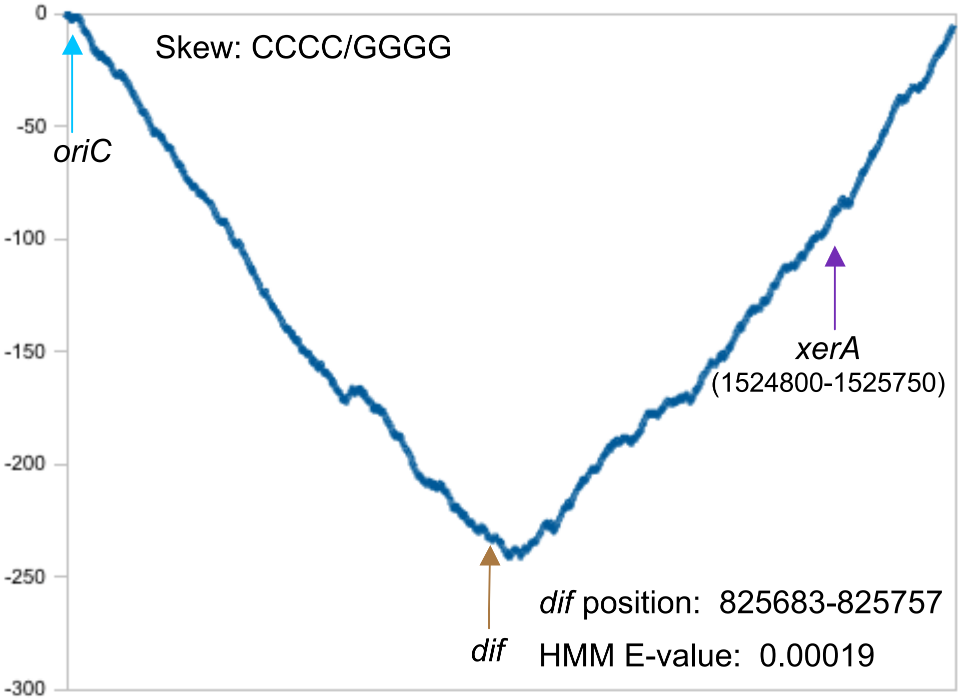

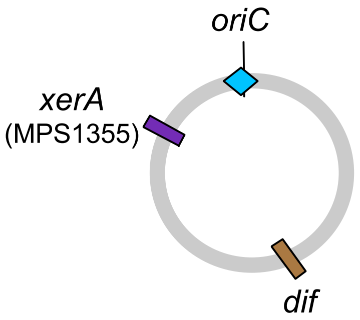

Sequences

***Thermococcales dif* sites**

	Conserved flanking sequence	Left Arm	Spacer	Rigth Arm	Conserved flanking sequence
P. horikoshii	ATGTTCATTTTTATACATTAC	TTTAGATATAA	TCAGCC	TTATATCTAAA	G TAATGTATAAAAATGAACATCAA
P. furiosus	ATGCTCATCTATGTTCATTAT	TTTAGATATAA	TCAGCC	TTATATCTAAA	A TCATGCACAAAAATGAGCGTCGA
P. abyssi	ATGCACATATTTGTGCGTTTA	ATTGGATATAA	TCGGCC	TTATATCTAAA	A TCATGAACAAAAATGAACGTTGA
T. kodakaraensis	GTGCCCATTTTTGTACTGCAGT	TTTTGATATAA	TGTACC	TTATATGACAA	ACTAATGAACAATAGCGAGCATAGA
T. onnurineus	ATGTTCAAACATGTTCGTGAAT	TTTAGATATAA	TGTGTC	TTATATCTAAA	ATTTATGTACATTGTTGTATATTGA
T. gammatolerans	ATGCTCGGACTTGATCACTAGT	TTTGGATATAA	TGATCC	TTATATCTAAA	GTCCCCGTACAGATATGAACATACA
T. sibiricus	TTGATTTTACCACTATACATAA	TTGTCATATAA	TAAGCC	TTATATCTAAA	ACTCATGCACAATAACATACATCAT
M.stadtmanae	ATCCTATTACTTGTAAATTTTT	ATTAGTTATAT	TATACA	AAATATCGACA	AATATTTAATAATTATAAACATATA
