# Supplementary material for: Evidence for a Xer/dif System for Chromosome Resolution in Archaea
Source: PLoS Genet. 2010 Oct 21;6(10):e1001166. doi: 10.1371/journal.pgen.1001166 (PMC2958812; doi:10.1371/journal.pgen.1001166)

Figure S7

Sulfolobales dif candidates (all within intergenic regions))

***dif* candidates**

		Left Arm	Spacer	Rigth Arm	Conserved flanking sequence	Genomic position	*xerA* gene	start	end
S. acidocaldarius	1	T T T G A G T T A A C	T C C C C A	G T T T A C T G A G A	A A A A A T T C A A C T	1 238 594	*Saci_1490*	1 267 333	1 268 196
S. tokodaii	1	A T T C A G T T A A C	T C C C C A	G T T T A C T G A G A	A A A A T T C T A T T T	1 313 131	*ST1393*	1 397 455	1 398 309
S. solfataricus	1	A T T G A G T T A A C	T C C C C G	G T T A A C T A A G A	A A A A C T T T A T A T	441 160	*SSO0375*	318 365	319 240
	2	A T T C A G T T A A C	T C C T C A	G T T T A C T G A G A	A A A A C T G T T T T T	479 363			

Skews: CACC/GGTG

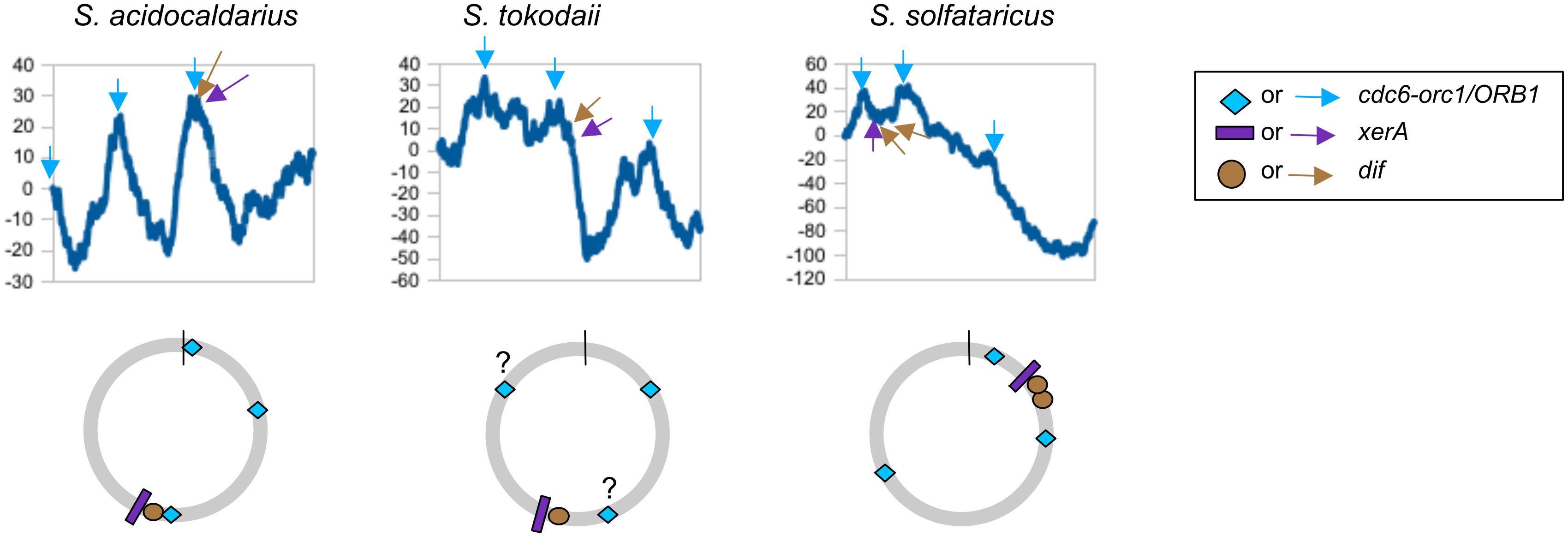

Supplement: Figure S7 — Sulfolobales dif sites. By using the methodology described in the main text of this article on S. solfataricus, S. acidocaldarius and S. tokodaii genomes, one single sequence that fits all of the requirements (two inverted repeats separated by a spacer of 4–8 base pairs, highly conserved between the three genomes and located inside intergenic regions) was found. This potential dif candidate is present only once in S. acidocaldarius and S. tokodaii, but has two copies (only one highly conserved), at the same chromosomal location in S. solfataricus genome. (0.10 MB PDF) [file pgen.1001166.s007.pdf]
