# Supplementary material for: Evidence for a Xer/dif System for Chromosome Resolution in Archaea
Source: PLoS Genet. 2010 Oct 21;6(10):e1001166. doi: 10.1371/journal.pgen.1001166 (PMC2958812; doi:10.1371/journal.pgen.1001166)
Supplement: Table S1 — Specific genomic positions of oriC-cdc6, xerA genes and dif sites in Thermococcales genomes. (0.06 MB PDF) [file pgen.1001166.s010.pdf]

**Table S1**

**Specific genomic positions of *oriC-cdc*6, *xerA* genes and *dif* sites in *Thermococcales* genomes**

| | ***oriC-cdc6*** | ***xerA*** | ***dif*** | ***dif* position from *oriC*** |
|---|---|---|---|---|
| ***P. abyssi*** | 121 402 | 372 604 | 1 220 236 | -142° |
| ***P. horikoshii*** | 109 476 | 1 587 912 | 736 553 | 135° |
| ***P. furiosus*** | 16 236 | 1 724 035 | 659 548 | 122° |
| ***T. kodakaraensis*** | 1 712 158 | 677 309 | 483 614 | 130° |
| ***T. onnurineus*** | 1 508 116 | 935 913 | 854 750 | -127° |
| ***T. gammatolerans*** | 125 431 | 1 541 676 | 1 457 016 | -132° |
| ***T. sibiricus*** | 13 000 (*oriC*)<br>1 434 100 (*cdc6*) | 1 110 121 | 689 100 | 132° |
